# The evolutionary history of the SAL1 gene family in eutherian mammals

**DOI:** 10.1186/1471-2148-11-148

**Published:** 2011-05-28

**Authors:** Camille Meslin, Fanny Brimau, Patricia Nagnan-Le Meillour, Isabelle Callebaut, Géraldine Pascal, Philippe Monget

**Affiliations:** 1 UMR85 Physiologie de la Reproduction et des Comportements, INRA, Nouzilly, F-37380, France; 2 UMR6175, CNRS, Nouzilly, F-37380, France; 3 Université François Rabelais de Tours, Tours, F-37041, France; 4 Haras Nationaux, Nouzilly, F-37380, France; 5 Unité de Glycobiologie Structurale et Fonctionnelle, INRA, UMR 8576 CNRS/Université Lille1, Villeneuve d'Ascq Cedex, F-59655, France; 6 IMPMC, UMR7590, CNRS, Université Pierre et Marie Curie, Paris, 75005, France

## Abstract

**Background:**

SAL1 (salivary lipocalin) is a member of the OBP (Odorant Binding Protein) family and is involved in chemical sexual communication in pig. SAL1 and its relatives may be involved in pheromone and olfactory receptor binding and in pre-mating behaviour. The evolutionary history and the selective pressures acting on SAL1 and its orthologous genes have not yet been exhaustively described. The aim of the present work was to study the evolution of these genes, to elucidate the role of selective pressures in their evolution and the consequences for their functions.

**Results:**

Here, we present the evolutionary history of SAL1 gene and its orthologous genes in mammals. We found that (1) SAL1 and its related genes arose in eutherian mammals with lineage-specific duplications in rodents, horse and cow and are lost in human, mouse lemur, bushbaby and orangutan, (2) the evolution of duplicated genes of horse, rat, mouse and guinea pig is driven by concerted evolution with extensive gene conversion events in mouse and guinea pig and by positive selection mainly acting on paralogous genes in horse and guinea pig, (3) positive selection was detected for amino acids involved in pheromone binding and amino acids putatively involved in olfactory receptor binding, (4) positive selection was also found for lineage, indicating a species-specific strategy for amino acid selection.

**Conclusions:**

This work provides new insights into the evolutionary history of SAL1 and its orthologs. On one hand, some genes are subject to concerted evolution and to an increase in dosage, suggesting the need for homogeneity of sequence and function in certain species. On the other hand, positive selection plays a role in the diversification of the functions of the family and in lineage, suggesting adaptive evolution, with possible consequences for speciation and for the reinforcement of prezygotic barriers.

## Background

The barriers that lead to divergence of species during the course of evolution were classified by Dobzhansky in two categories: prezygotic and postzygotic reproductive barriers [1]. Postzygotic reproductive barriers concern all the events that occur after fertilization, such as reduced hybrid viability and fertility, while prezygotic reproductive barriers concern isolation of sexual partners via ecological, temporal or behavioral isolation. Pheromones play a key role in pre-mating recognition of sexual partners [2]. These compounds are defined as substances released by an animal that are able to induce specific behavioral and/or endocrinological reactions in a sexual partner of the same species [3]. Through these reactions, they could be involved in mate choice and sexual selection.

Odorant binding proteins (OBP) are small soluble proteins that are present in the olfactory apparatus as well as in biological fluids such as saliva, urine or vaginal discharge, and are able to bind pheromones (for review see [4]). OBP are assumed to be directly involved in chemical communication and in the pre-mating recognition process. Three hypotheses are proposed concerning their mechanism of action. The first is that olfactory receptors can recognize the OBP/pheromone complex, not just the pheromone alone. The second hypothesis is that the pheromone can be transferred to olfactory receptors only if assisted by the OBP. The third hypothesis is that the ligand can spontaneously dissociate from the complex with OBP and bind to the receptor as a "free pheromone" [5].

The role of saliva in chemical communication between males and females is well established in pig [6], like the role of urine in mouse [7]. In pig, saliva contains the pheromonal steroids 5α-androst-16-en-3-one and 5α-androst-16-en-3α-ol, as well as abundant quantities of salivary lipocalin (SAL1), the most abundant OBP isolated from submaxillary glands of mature males. When extracted from its source, this protein is associated with both pheromonal steroids [8], and appears to play a key role in the standing reflex in the sow [6] and also in the boar's libido [9]. SAL1 is also expressed in the nasal and vomeronasal area, but devoid of ligand [10,11]. SAL1 exhibits a classical structure of lipocalins characterized by a fully conserved N-terminal -G-X-W- motif and the typical folding pattern of a nine-stranded antiparallel β-barrel forming an internal ligand binding site for small hydrophobic molecules [12], despite relatively low sequence similarity [13]. SAL1 also possesses a glycosylation site on Asn53. Two natural variants have been identified in which in three residues differ (Val61, Ile64 and Ala89 of isoform A are respectively Ala, Val and Val in isoform B). Two residues (Val61 and Ala89) are located inside the β-barrel while the third residue (Ile64) is located next to the β-barrel, suggesting that these minor structural differences lead to ligand binding specificities [14].

Olfactory receptors are located on the olfactory sensory neurons of the main olfactory system in mammals and on the vomeronasal organ in rodents and other non-primate species [15]. Several authors examined the evolution of olfactory receptors, but few studies of lipocalins and OBP have been performed. Ganfornina et al. [16] undertook phylogenetic analysis of prokaryotic and eukaryotic lipocalins and showed that this family appeared early and is composed of 13 monophyletic clades. These authors also showed that ancestral lipocalin clades in the phylogenetic tree are able to bind large ligands while more recent lipocalin clades, such as clades composed of OBP and MUP (Mouse Urinary Protein), bind smaller ligands. They also found that later clades had higher rates of amino acid substitution, more flexible protein structures and greater ligand-binding efficiency than more ancestral lipocalins.

Logan et al. [17] undertook an extensive study of the *Mup *cluster in the mouse genome. They identified 21 *Mup *genes and 21 *Mup *pseudogenes on chromosome 4. They also identified *Mup *gene expansion in rat (9 genes and 13 pseudogenes), in horse (3 genes) and in mouse lemur (2 genes and 1 pseudogene) in the same syntenic region. Orangutan, chimpanzee, dog, pig (with SAL1), bushbaby and rhesus monkey have only one *Mup *gene in the syntenic region. The inferred phylogeny, the accumulation of synonymous substitutions, and the genomic organization of the *Mup *loci suggest that gene expansion occurred independently in several species [17].

In the light of previous analyses, the aim of the present work was to study the evolution of SAL1 which is involved in pre-mating recognition in pig. We wanted to determine if selective pressures act on these proteins and to check if positive selection may play a role in binding specificity toward ligands or olfactory receptors.

## Results

### Identification of SAL1 homologous genes, genomic localization and phylogenetic study

**Figure 1:**
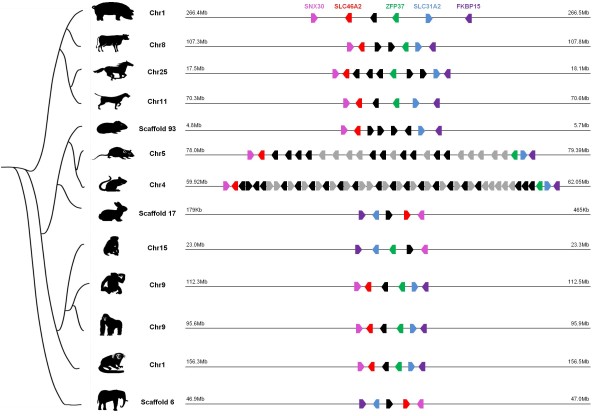
**Lineage specific expansion of the SAL1 family**. Genomic localization of SAL1 and its orthologous genes in 13 species: pig (*Sus scrofa*), cow (*Bos taurus*), horse (*Equus caballus*), dog (*Canis familiaris*), guinea pig (*Cavia porcellus*), rat (*Rattus norvegicus*), mouse (*Mus musculus*), rabbit (*Oryctolagus cuniculus*), macaque (*Macaca mulatta*), chimpanzee (*Pan troglodytes*), gorilla (*Gorilla gorilla*), marmoset (*Callithrix jacchus*) and elephant (*Loxodonta Africana*). Genes that belong to SAL1 family are in black. Pseudogenes are in gray. Orthologous flanking genes SNX30, SLC46A2, ZFP37, SLC31A2 and FKBP15 are in pink, red, green, blue and purple, respectively. Numbers of chromosomes and localization of genes on chromosomes are indicated near each species.

**Figure 2:**
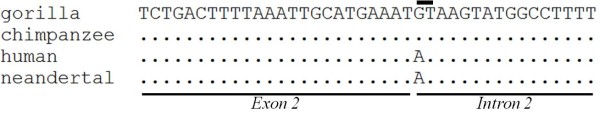
**Loss of SAL1 orthologous gene in human and Neandertal genomes**. The underlined A represents the G-to-A substitution in the donor site of the second intron in human and Neandertal genomic sequences, resulting in a shift in the ORF and in the pseudogenization of the gene in the two species.

**Figure 3:**
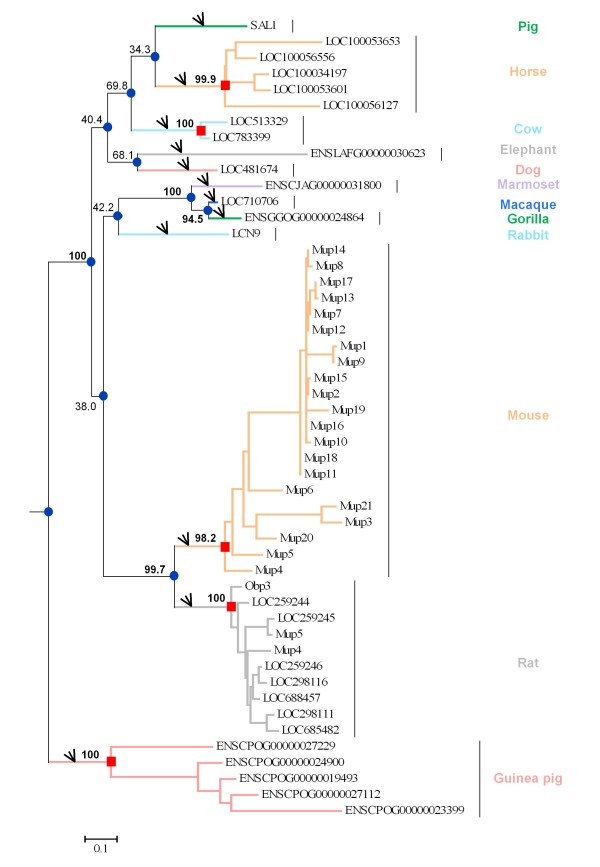
**Phylogenetic tree of SAL1 family**. The phylogenetic tree was reconstructed using maximum likelihood (ML) and rooted by midpoint rooting. Bootstrap values are given for main branches, in bold when nodes are strongly supported (>80%). The 50 mammalian SAL1 amino-acid sequences related to the 50 identified functional genes were used to reconstruct the phylogenetic tree. After removal of gaps, the dataset comprised 119 sites. Blue circles represent speciation and red squares duplication. Branches on the tree that were tested for positive selection on species clades are indicated by black arrows.

We found similar sequences to pig SAL1 in 13 other mammalian species: cow, horse, dog, guinea pig, rat, mouse, rabbit, macaque, chimpanzee, gorilla, marmoset and elephant (Figure 1 and table S1 in additional file 1). The sequences identified were located at the same syntenic locus between the neighboring genes SLC46A2 and ZFP37 and form the SAL1 family. Putative pseudogenes were identified in mouse, rat, mouse lemur, bushbaby and orangutan (Figure 1 and figure S1 in additional file 1). The exact number of genes we identified in mouse, rat, chimpanzee, guinea pig, cow, mouse lemur, orangutan and bushbaby differed from that found by Logan et al. [17], who failed to identify several of these genes in a previous study, probably due to lower quality genome annotation. Mouse and rat genes form two important clusters of duplicates on chromosome 4 and 5, respectively, composed of 21 genes and 21 pseudogenes in mouse, and 10 genes and 11 pseudogenes in rat. Duplicates are present in cow (2 genes), horse (5 genes) and guinea pig (5 genes). All genes of each species form a cluster, suggesting cis-duplication events after speciation. Pig, dog, rhesus monkey and chimpanzee possess a single gene similar to SAL1 in the same syntenic region. In human, the gene is a known pseudogene [18] due to a G-to-A nucleotide substitution at the donor site of the second intron, resulting in the split of the ORF of the coding sequences. This substitution was not found in chimpanzee and other primates. In the Neandertal Genome [19], we found the same genomic organization as in human in ENSEMBL [20], namely the two genes SLC46A2 and ZFP37 surrounding a predicted SAL1 pseudogene. After multiple sequence alignment of this genomic region between chimpanzee, gorilla, Neandertal and human, we found the same substitution in the Neandertal genome, suggesting the emergence of this mutation in the common ancestor of Neandertal and human (Figure 2). The SLC46A2/ZFP37 locus is not present in frog (*Xenopus tropicalis*), birds (*Gallus gallus*, *Taeniopygia guttata*), bony fishes (*Takifugu rubripes, Tetraodon nigroviridis, Gasterosteus aculeatus, Danio rerio *and *Oryzias latipes)*, monotremes (*Ornithorhynchus anatinus*) and marsupials (*Monodelphis domestica)*, suggesting this family emerged in eutherian mammals. Orthology and paralogy relationships between the identified genes were inferred from the phylogenetic tree (Figure 3). Monophyletic clades formed by genes belonging to the same species were supported by very high bootstrap values (94.5 to 100%), suggesting that gene duplications occurred independently in mouse, rat, guinea pig, horse, and cow. The relationships between some species were not clear because of low bootstrap values for some nodes (34.3 to 68.1%), even if rodents and primate clades were supported by high bootstrap values (99.7 and 100%, respectively). The percentage of identity between sequences of this family is highly variable, not only between species but also between paralogs. In mouse, for example, Mup11 and Mup18 amino acid sequences are strictly identical. In rat, some paralogs are more distinct and pairwise identity ranges from 84 to 97%, so we tested paralog datasets for gene conversion events.

### Evolution of paralogs in the SAL1 family

Gene conversion can occur between paralogous regions if they have sufficient sequence identity. To determine whether the identified clusters of paralogs underwent gene conversion events, we searched for statistical evidence of this phenomenon using the GENECONV program [21]. The first control analysis of "Randomize sites", which randomizes the order of polymorphic sites before analysis, detected no gene conversion event in horse, rat, mouse and guinea pig datasets, implying the results of subsequent GENECONV analyses are reliable. As shown in Table 1, most of the paralogs of guinea pig and mouse are involved in gene conversion events while in horse and in rat, respectively 3 and 4 genes out of a total of 5 and 10 are involved. The length of the converted tract varied greatly among species, from 11 bp for the shortest tract in guinea pig, to 529 bp for the longest tract in mouse. To determine which type of selective pressure (positive, neutral or purifying selection) shaped the evolution of these genes after gene duplication, we assessed selective pressure using the nonsynonymous/synonymous substitution rate ratio (ω) with codon-substitution models, where ω< 1 is purifying selection, ω = 1 is neutral evolution and ω > 1 is consistent with positive Darwinian selection [22,23]. We performed a branch-site-based analysis by defining each branch supporting a paralogous gene as a foreground branch for PAML. In each species where the SAL1 gene has been duplicated, only one gene underwent positive selection (Table 2). Significant Likelihood Ratio Tests (LRTs) were found for the five genes, confirming that a positive selection model fits the data. For cow, mouse and rat genes, only a few (one or two) positively selected sites were detected, whereas in horse and particularly in guinea pig, more positively selected sites (6 and 15 sites, respectively) were detected.

**Table 1:** Interlocus gene conversion events

Species	**Number of sequences in the dataset **^**(1)**^	**Number of sequence involved in a gene conversion event **^**(2)**^	Converted tract length (bp)	
			
			min	max
Horse	5	3	57	74

Rat	10	4	161	273

Mouse	21	20	107	529

Guinea pig	5	5	11	135

^(1) ^All sequences are indicated in the table S1 of the additional file 1.

^(2) ^Sequences involved in gene conversion events are indicated in bold in the table S1 of the additional file 1.

**Table 2:** Parameter estimates and likelihood scores for branch-site models for paralogs

Genes	Model	***l ***^**(1)**^	**Estimates of parameters **^**(2)**^	**2Δ*l ***^**(3)**^	Positively selected sites (BEB)
Cow LOC783399	Null	-5660.941163	ρ0 = 0.32, (ρ1 = 0.68), ω0 = 0.24, (ω1 = 1)	27.10 ***	Not allowed
			
	Alternative	-5647.392083	ρ0 = 0.30, ρ1 = 0.66, (ρ2 = 0.05), ω0 = 0.23, (ω1 = 1), ω2 = ∞		1 site p > 99%: 36Y

Guinea pig ENSCPOG00000023399	Null	-5660.050892	ρ0 = 0.25, (ρ1 = 0.50), ω0 = 0.24, (ω1 = 1)	56.40 ***	Not allowed
			
	Alternative	-5631.851037	ρ0 = 0.27, ρ1 = 0.55, (ρ2 = 0.18), ω0 = 0.23, (ω1 = 1), ω2 = 336.70		7 sites p > 99%: 24R, 27A, 113L, 118Q, 123T, 125T, 128T, 8 sites p > 95%: 31L, 25E, 26T, 85V, 114T, 117T, 122V, 126L

Horse LOC100053653	Null	-5665.171131	ρ0 = 0.22, (ρ1 = 0.47), ω0 = 0.23, (ω1 = 1)	15.86 ***	Not allowed
			
	Alternative	-5657.239232	ρ0 = 0.09, ρ1 = 0.19, (ρ2 = 0.72), ω0 = 0.23, (ω1 = 1), ω2 = 6.44		6 sites p > 95%: 81E, 97A, 123Q, 135K, 166K, 168F

Mouse Mup19	Null	-5660.941163	ρ0 = 0.32, (ρ1 = 0.67), ω0 = 0.24, (ω1 = 1)	53.87 ***	Not allowed
			
	Alternative	-5634.004112	ρ0 = 0.30, ρ1 = 0.66, (ρ2 = 0.04), ω0 = 0.23, (ω1 = 1), ω2 = ∞		2 sites p > 99%: 174K, 177F

Rat LOC298116	Null	-5660.941163	ρ0 = 0.32, (ρ1 = 0.67), ω0 = 0.24, (ω1 = 1)	8.69 ***	Not allowed
			
	Alternative	-5656.597218	ρ0 = 0.32, ρ1 = 0.66, (ρ2 = 0.02), ω0 = 0.24, (ω1 = 1), ω2 = ∞		1 site p > 99%: 135A

^(1) ^Log- likelihood values.

^(2) ^ρ0, ρ1, and ρ2 are the proportions of codons subject to purifying selection, neutral evolution, and positive selection, respectively. ω0, ω1 and ω2 represented dN/dS for each class (purifying, neutral and positive selection, respectively).

^(3) ^*** significant at *p *< 0.001.

### Positively selected sites in the SAL1 family and putative biological significance

To identify the selective pressure on the SAL1 family in eutherian mammals, we performed a site-based analysis with PAML (Table 3). After removal of gaps, 119 sites were analyzed using the codeml [24] and Selecton [25] programs. In both comparisons (M1a vs. M2a, M8a vs. M8), LRTs were significant (*p *< 0.001) for the dataset. Moreover, the AIC_c _score of MEC was lower than that of M8a, indicating that MEC fits the data better. Comparisons of the LRT and AICc scores were significant, implying that selective forces varied among sites between genes. According to M2a and M8 models, 23 to 29% of sites underwent positive selection, respectively. Four sites (9V, 72Y, 73A and 90E) were identified as positively selected sites with a p-value of at least 95% by the three models (M2a, M8 and MEC), a strong indication of positive selection for these four amino acids. Six sites (10T, 62R, 75C, 86A, 159R and 162Q) were identified by the M2a and M8 models. Six sites (6Q, 11S, 63K, 71F, 113G and 119L) were identified by M8, and one site (163L) was identified by MEC.

**Table 3:** Parameter estimates and likelihood scores for site models

Model	***l ***^**(1)**^	**Estimates of parameters **^**(2)**^	**2Δ*l ***^**(3)**^	**Positively selected sites (BEB) **^**(4)**^
M0	-5729.43479	ω = 0.92		Not allowed

M1a	-5660.94116	ρ0 = 0.32		Not allowed

M2a	-5643.14631	ρ0 = 0.24, ρ1 = 0.53, ρs = 0.23, ωs = 2.08	35.59 *** (M2a vs M1a)	1 site p > 99%: **72Y**, 9 sites p > 95%: **9V**, *10T*, *62R*, **73A**, *75C*, *86A*, **90E**, *159R*, *162Q*

M7	-5661.09613	p = 0.64, q = 0.24		Not allowed

M8a	-5657.82124	ρ0 = 0.41, ρ1 = 0.59, p = 1.69, q = 3.62		Not allowed

M8	-5641.30866	ρ0 = 0.71, ρs = 0.29, p = 0.83, q = 0.44, ωs = 1.84	33.02 *** (M8 vs M8a)	9 sites p > 99%: **9V**, *10T*, *62R*, **72Y**, **73A**, *75C*, *86A*, **90E**, *162Q*; 7 sites p > 95%: 6Q, 11S, 63K, 71F, 113G, 119L, *159R*

**Model**	**AICc score**	**Estimates of parameters (2)**		**Positively selected sites (BEB) (4)**

M8a	18637.95571	p = 1.10, q = 1.73		Not allowed

MEC	18220.66368	p = 0.80, q = 2.56		5 sites p > 95%: **9V**, **72Y**, **73A**, **90E**, 163L

^(1) ^Log- likelihood values.

^(2) ^ωS: average dN/dS ratio for sites subject to positive selection (models M2a and M8), p and q: shape parameters for the beta distribution of ω (models M7, M8 and MEC). ρ0, ρ1, and ρS are the proportions of codons subject to purifying selection, neutral evolution, and positive selection, respectively.

^(3) ^*** significant at *p *< 0.001.

^(4) ^Bold: *P *> 95% for the three comparisons (M2a vs. M1a, M8 vs. M8a, MEC vs. M8a).

Italic: *P *> 95% for the two comparisons (M2a vs. M1a and M8 vs. M8a).

Underlined: amino acids involved in androstenol and androstenone binding.

Site numbers and amino acids refer to the pig SAL1 reference sequence PDB: 1GM6.

**Figure 4:**
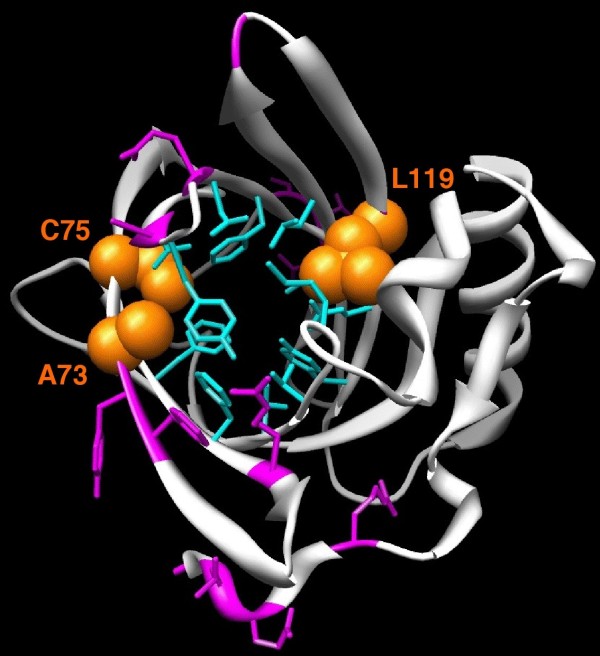
**Positive selection acting on orthologs**. Map of the amino acids involved in the binding of ligand [13] on the SAL1 3D structure are in blue, and positively selected sites in pink (PDB: 1GM6). Amino acids shown in a van der Waals representation (orange) were both involved in ligand binding and were positively selected. Positively selected amino acids were identified by PAML computations using site models.

**Figure 5:**
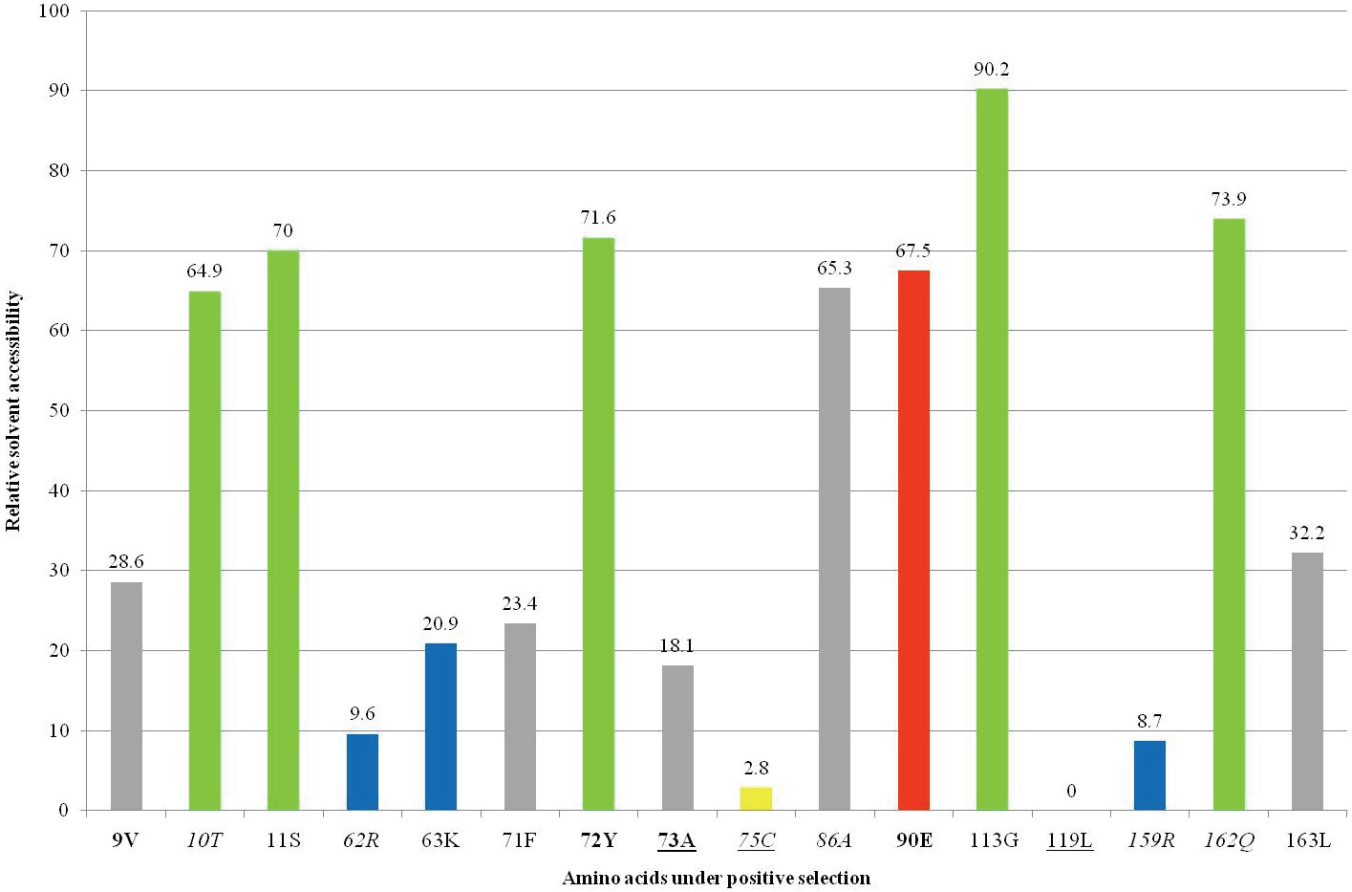
**Positively selected sites and solvent accessibility**. Positively charged, negatively charged, polar and non-polar residues are in blue, red, green and gray respectively, the same as in the ASAView. Bold: amino acids with *P *> 95% for the three pairs of comparison (M2a vs. M1a, M8 vs. M8a, MEC vs. M8a). Italics: amino acids with *P *> 95% for the two pairs of comparison (M2a vs. M1a and M8 vs. M8a). Underlined: amino acids involved in androstenol and androstenone binding.

To determine if positively selected sites are located in regions of interest, these sites were mapped on the 3D structure of SAL1 (PDB:1GM6) (Figure 4). To assess the biological significance of these sites, ligand binding sites determined by Spinelli et al. [13] were also mapped on the 3D structure. Interestingly, three sites under positive selection matched amino acids that are directly involved in androstenol and androstenone binding (73A, 75C and 119L). Side chains of amino acids involved in ligand binding projected into the ligand binding pocket, which is formed by a relatively small internal cavity poorly accessible to solvent, whereas side chains of the majority of positively selected sites projected out of the binding pocket, except for three amino acids, suggesting that positive selection does not only play a role in pheromone binding specificity, but also in interaction with partners such as receptors. Relative solvent accessibility (RSA) of positively selected sites was determined by ASAView and is shown in Figure 5. We used the same classification as Rost et al., [26]: a residue is classified as buried when the RSA is <9%, as exposed when the RSA is >35%, and as intermediate when the RSA is between 9 and 35%. We found three buried sites (75C, 119L and 159R), six intermediate sites (9V, 62R, 63K, 71F, 73A and 163L) and seven exposed sites (10T, 11S, 72Y, 86A, 90E, 113G and 162Q), indicating that most of the positively selected sites are located at the surface of the protein, and are perhaps involved in other functions than pheromone binding, however these remain to be identified. We observed no specific clustering of these sites at the surface of the protein exposed to the solvent (Figure 4).

### Positive selection events in marmoset, dog, guinea pig, horse and mouse clades

**Figure 6:**
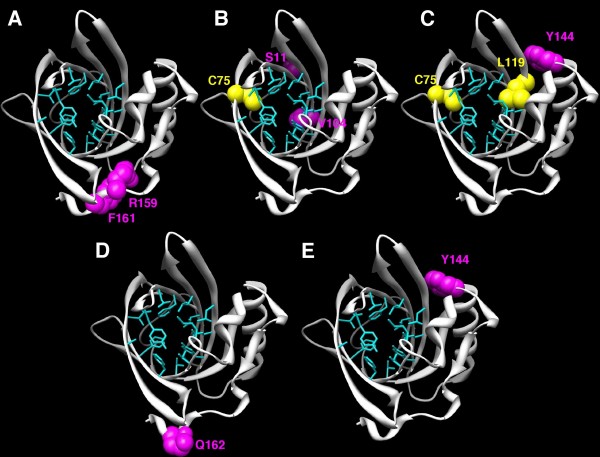
**Positive selection acting on species clades**. Positively selected sites identified on species clades are shown in a van der Waals representation on the SAL1 3D structure. Positively selected sites that matched amino acids involved in ligand binding are in yellow, the others in pink. Amino acids involved in ligand binding are in blue, the same as in Figure 4. Positively selected amino acids were identified by PAML computations using branch site models. A: in marmoset, B: in guinea pig, C: in horse, D: in mouse and E: in dog.

The comparison between site models of PAML detects positive selection only if the ω ratio averaged over all branches on the tree is greater than 1, but positive selection can also be expected to affect only a few amino acid residues in certain lineages. For this reason, we used branch-site models [27] that are designed to detect signals of local episodic positive selection in order to determine whether different species underwent selective pressure. We tested the 12 species clades as foreground branches with branch-site models of PAML. The branches tested are shown in Figure 3. We were unable to draw any conclusions concerning cow, gorilla, elephant, macaque, rat and pig clades, as the LRTs were not significant. We found significant LRTs in the marmoset, dog, guinea pig, horse, mouse and rabbit clades, suggesting that SAL1 orthologs underwent positive selection in these species, but we were unable to identify any selected sites in rabbit (Table 4). For the other species, positively selected sites were mapped on the SAL1 structure. Mapping of site 14H identified in dog was not possible because this amino acid is situated at the beginning of the N-terminal end, which was not crystallized. Sites identified in marmoset, mouse and dog were not located in the binding pocket of the protein unlike some sites identified in guinea pig and horse. For the two latter clades, one (75C) and two (75C and 119L) positively selected sites, respectively, matched pheromone binding sites (Figure 6).

**Table 4:** Parameter estimates and likelihood scores for branch- site models for 5 species

Species	Model	***l ***^**(1)**^	**Estimates of parameters **^**(2)**^	**2Δ*l ***^**(3)**^	Positively selected sites (BEB)
Marmoset	Null	-5660.37202	ρ0 = 0.18, (ρ1 = 0.39), ω0 = 0.23, (ω1 = 1)	18.47 ***	Not allowed
			
	Alternative	-5651.1375	ρ0 = 0.30, ρ1 = 0.63, (ρ2 = 0.07), ω0 = 0.23, (ω1 = 1), ω2 = 60.38		2 sites p > 95%: 159R, 161F

Dog	Null	-5660.55998	ρ0 = 0.25, (ρ1 = 0.52), ω0 = 0.24, (ω1 = 1)	6.78 **	Not allowed
			
	Alternative	-5657.16845	ρ0 = 0.28, ρ1 = 0.58, (ρ2 = s0.14), ω0 = 0.23, (ω1 = 1), ω2 = 11.15		2 sites p > 95%: **14H**, 144Y

Guinea pig	Null	-5660.94116	ρ0 = 0.32, (ρ1 = 0.67), ω0 = 0.24, (ω1 = 1)	13.68 ***	Not allowed
			
	Alternative	-5654.10045	ρ0 = 0.27, ρ1 = 0.51, (ρ2 = 0.22), ω0 = 0.25, (ω1 = 1), ω2 = 3.94		3 sites p > 95%: 11S, 75C, 104V

Horse	Null	-5660.94121	ρ0 = 0.32, (ρ1 = 0.67), ω0 = 0.24, (ω1 = 1 )	27.22 ***	Not allowed
			
	Alternative	-5647.32999	ρ0 = 0.30, ρ1 = 0.61, (ρ2 = 0.09), ω0 = 0.24, (ω1 = 1), ω2 = 7.57		2 sites p > 99%: 75C, 119L; 1 site p > 95%: 144Y

Mouse	Null	-5658.60187	ρ0 = 0.24, (ρ1 = 0.55), ω0 = 0.19, (ω1 = 1 )	10.75 **	Not allowed
			
	Alternative	-5653.22783	ρ0 = 0.25, ρ1 = 0.58, (ρ2 = 0.17), ω0 = 0.19, (ω1 = 1), ω2 = 3.25		1 site p > 95%: 162Q

^(1) ^Log- likelihood values.

^(2) ^ρ0, ρ1, and ρ2 are the proportions of codons subject to purifying selection, neutral evolution, and positive selection, respectively. ω0, ω1 and ω2 represented dN/dS for each class (purifying, neutral and positive selection, respectively).

^(3) ^** significant at *p *< 0.01

*** significant at *p *< 0.001.

^(4) ^Underlined: amino acids involved in androstenol and androstenone binding.

Site numbers and amino acids refer to the pig SAL1 reference sequence PDB: 1GM6, except for the amino acid in bold, which is not part of the structure but comes from the dog genome sequence XP_855342.1.

## Discussion

Phylogenomic analyses showed that the SAL1 family originated in eutherian mammals and that genes belonging to this family were duplicated after speciation events in five mammalian species. In certain living species, such as mouse lemur, bushbaby, orangutan and human, the gene has been lost, as it has in the Neanderthal genome. We can date the loss of the gene in hominid before the Neandertal-modern human split, 400,000 to 350,000 years ago [28]. The number of duplication events varies greatly among species. In mouse and rat, massive cis-duplication events have occurred, with respectively 42 and 22 genes in the cluster, followed by gene loss with respectively 21 and 11 pseudogenes.

Gene duplication represents a source of new genetic material, and can lead to evolutionary novelties. The fate of duplicated genes can follow different models of evolution, with different selective pressures acting on the genes [29]. We checked for a change in selective pressure in all paralogs identified in the SAL1 family and found that only a few paralogs underwent positive selection: one gene in cow, guinea pig, horse, mouse and rat. Moreover, few sites of these genes were identified. A large proportion (66%) of each gene evolved under neutrality and only a small proportion (2 to 5%) under positive selection. However, among the single genes identified as positively selected in guinea pig and horse, a larger proportion of sites evolved under positive selection (18 and 72%, respectively) and more sites were identified as being positively selected (15 sites in guinea pig and 6 sites in horse).

Because sequences of some paralogs share high similarity, we searched for gene conversion in our paralogous gene datasets and found extensive interlocus gene conversion events in mouse and guinea pig, and to a lesser extent in horse and rat. Karn and Laukaitis [30] compared the mouse *Mup *cluster with a gene tree published by Mudge et al. [31] and suggested that concerted evolution masked the common origin of the gene and neighboring pseudogenes [30]. Our results confirmed this hypothesis, indicating extensive gene conversion in the mouse *Mup *cluster. This extensive gene conversion phenomenon led to sequence homogenization and is the cause of the concerted evolution of these genes. Such extensive concerted evolution suggests that, at least in mouse and guinea pig, both maintenance of sequence homogeneity and increased gene dosage are important for these species. The evolution of SAL1 paralogs resembles the evolution of the β-globin gene family. In this family, paralogous copies evolved under a process of functional divergence and there is evidence for two gene conversion events in mouse and goat clusters composed of β-globin duplicated genes There is also evidence for variable selective pressure among sites for β and γ-globin genes with 4 to 9% of sites evolving under positive selection [32].

By combining phylogenetic, gene conversion and selective pressure results on paralogs evolution, we can try to describe the fate of duplicated genes, in which duplication can be seen as an advantageous phenomenon for the species concerned, by combining two scenarios from Innan and Kondrashov [29]. In the first scenario, one could consider the massive duplication in rat and mouse as a gene amplification where the increase in dosage of these genes is beneficial. This scenario of evolution corresponds to category IIa described by Innan and Kondrashov [29]. In this model, if selection for the duplicated copy is weak, pseudogenization can occur if a null mutation is fixed, which is the case in both mouse and rat. The occurrence of gene conversions that maintain sequence similarity and promote conservation of gene copies could be consistent with that hypothesis, but the high frequency of gene conversion events is not restricted to mouse and rat. In fact, guinea pigs, which do not harbor large gene amplifications, have the highest frequency of conversion events per gene copy among the species tested. The beneficial increase in dosage has already been shown to apply to genes that mediate the interaction between the organism and the environment [33], as is true of genes of the SAL1 family. However, we also showed that among the many duplicates in rat, mouse and guinea pig, one gene per species is under positive selection so increased gene dosage and gene conversion events are not the only driving force of the evolution of these genes in these species. For these positively selected duplicates, it is the scenario of the category III [29] which fits, where a new copy can be fixed and preserved by positive selection, leading to the possible emergence of a new function for the positively selected gene.

To study selective pressure in the SAL1 family in more detail, we tested the amino acids changing occurring in the 12 branches supporting species as positively selected by PAML. Our results showed that marmoset, dog, guinea pig, horse and mouse branches underwent positive selection just after divergence. This evolutionary scenario likely reflects the ability of the SAL1 family to diverge and to adapt to new behaviour between sexual partners. A previous study on mouse and rat genes identified 32 sites as positively selected on rodent co-orthologs of SAL1 [34]. In that study, mouse and rat genes were considered together, whereas in our study, mouse and rat genes were analyzed separately, this explains the difference between the two results. Indeed, we only identified one site under positive selection in mouse and no positive selection in rat. The difference between the two results is also due to a difference in the probability threshold chosen to determine whether a site is subject to positive selection or not. In Emes et al. [34], a site was said to be positively selected if the probability for one model is > 0.90 and > 0.50 for at least one other model. In our study, we chose to consider only sites whose probability was > 0.95 in order to minimize false positives.

Finally, we compared tests of variable selective pressures for the family using several PAML codon models. We found evidence for positive selection in a small proportion of sites. Because positive selection is known to play a role in the diversification of protein functions, we mapped all positively selected sites on the 3D structure, in order to assess their biological significance for the gene family as a whole and for each species independently. Apart from the three amino acids that were under positive selection and involved in ligand binding, the other amino acids identified by site models of PAML analyses projected out of the binding pocket. Moreover, the majority of these sites were exposed to solvent. If these sites were involved in the interaction with pheromones, they would be found preferentially in the hydrophobic core and would be buried. We thus propose that positive selection plays a role not only in the binding specificity but also in the interaction between the protein and its environment. We were not able to draw any conclusions concerning selective pressures on each site involved in ligand binding, because gaps in the multiple sequence alignment made these calculations impossible. Nevertheless, for the 16 amino acids involved in pheromone binding, we identified three sites that probably evolved under purifying constraints (87Y, 91N and 93F) and four sites that probably evolved under relaxed constraints (60F, 85V, 121E and 123Y). The three sites that evolved under purifying constraints may be essential for protein function, because they were well conserved during the evolution of the family. In rodent populations, Emes et al. [34] found that MUPs, which are co-orthologs of SAL1, exhibited amino acids under positive selection, and that these positively selected sites were located at the interface between MUPs and their receptors, probably V2R receptors on the vomeronasal organ. They also found evidence that olfactory receptors, such as V2Rs, underwent positive selection. The hypothesis they proposed is that this adaptation phenomenon is due to conspecific competition, resulting in well adapted pheromones, pheromone binding proteins such as MUP, and olfactory receptors [34]. Our results allow us to extend this hypothesis because positive selection also drives the evolution of pheromone binding proteins in other eutherian mammals. So for all the family, and not just for rodents, there is an adaptive evolution of these proteins to their ligands and maybe their receptors, too. It would be interesting to test if V2R receptors are subject to positive selection, not only in rodents but also in other mammals. Several authors reported evidence for positive selection on other OR genes in mammals [35-39], with possible involvement of positively selected sites in the binding property of proteins. Moreno-Estrada [36] suggested that positive selection could be at the origin of a new ligand binding capability or the modification of odorant perception and could improve the overall degenerated OR gene repertoire, at least in human. In insects, co-evolution of the two enzymes involved in the pheromone biosynthetic pathway and in the pheromone receptor has been suggested to play a role in the speciation process [40]. It would be interesting to test co-evolution of enzyme/receptor, pheromone/receptor and OBP/receptor in mammals.

In mice, MUPs are important for the delivery, via urine, of chemical signals conveying information about the sex and hormonal status of the animal who release the scent mark [41]. In pig, SAL1 may be involved in pre-mating recognition by binding pig specific sex pheromones in saliva [8]. In both species, these proteins are involved in conspecific recognition in the context of reproduction. When the genomes of marine mammals are completed, it will be interesting to search for SAL1 orthologs. Indeed, in such a different environment, chemical communication between sexual partners is probably not mediated by the same olfactory cues as in terrestrial mammals. If a SAL1 ortholog is found in marine mammal genomes, it will be interesting to discover if it evolved under relaxed constraints or positive selection.

It is well established that reproduction is a very competitive process, and that selective pressures on genes involved in the process are not rare (for a review, see [42]). Positive Darwinian selection is not atypical, especially for genes involved in sensory perception and mate choice [43]. Our results demonstrated that (i) positively selected sites differ between genes and (ii) positively selected sites are involved in ligand binding and are putatively involved in receptor binding. Such a selective pressure on these proteins could be at the origin of a divergence process between species and thus contribute to the speciation phenomenon by reinforcing prezygotic barriers. To test this hypothesis, we performed *in vitro *mutagenesis experiments on SAL1, but the poor folding of the resulting proteins prevented further experimentation.

## Conclusions

The SAL1 gene family originated in eutherian mammals and duplicated after speciation in cow, horse, guinea pig and rodents. Some duplicated genes underwent concerted evolution with extensive gene conversion. Others were subject to positive selection at different sites, and our knowledge of the 3D structure of this protein suggests that the selected sites are involved in pheromone binding and possibly in olfactory receptor binding. This result suggests a functional divergence between species because positively selected sites differ between species. All these data suggest that the evolution of the SAL1 family allows a species-specific strategy to transduce pheromonal signals in mammals, reinforcing species divergence through species-specific sexual behaviour.

## Methods

### Phylogenetic and syntenic analyses

The protein sequence of the pig salivary lipocalin (SAL1) was retrieved from GenBank (http://www.ncbi.nlm.nih.gov/genbank/) [44] (NP_998979.1). Proteins from other species were searched by using TBLASTN with porcine protein sequence as the query against all mammalian genomes available on the NCBI (http://www.ncbi.nlm.nih.gov/mapview/) [45] and ENSEMBL databases (http://www.ensembl.org/index.html) [46]. Identified proteins were then located on genomes for syntenic analyses of the most recent genome sequence assemblies: pig (*Sus scrofa*: ENSEMBL Sscrofa9), cow (*Bos Taurus*: NCBI Btau5.2), horse (*Equus caballus*: NCBI EquCab2.0), dog (*Canis familiaris*: ENSEMBL CanFam2.0), guinea pig (*Cavia porcellus*: ENSEMBL cavPor3), rat (*Rattus norvegicus*: NCBI RGSC 3.4), mouse (*Mus musculus*: NCBIM37), rabbit (*Oryctolagus cuniculus*: ENSEMBL OryCun2), rhesus monkey (*Macaca mulatta*: NCBI Build 1.2), chimpanzee (*Pan troglodytes*: NCBI Build 2.1), gorilla (*Gorilla gorilla*: ENSEMBL gorGor3), marmoset (*Callithrix jacchus*: ENSEMBL C_jacchus3.2.1) and elephant (*Loxodonta Africana*: ENSEMBL loxAfr3). To improve homology assignment, we only included genes from the same syntenic region in the final dataset. Sequences with no syntenic information were discarded. No genes were identified in other available mammalian genomes, and existing genome assemblies did not allow us to identify the syntenic region. Multiple sequence alignments were performed using the Clustal W algorithm [47]. The chimpanzee sequence was removed from the dataset in order to have the most possible informative sites. All alignment gap sites were removed before phylogenetic analyses. Phylogenetic trees were reconstructed using maximum likelihood (ML) in PhyML 3.0 [48] in order to establish orthologous and paralogous relationships among the gene datasets. Bootstrap values [49] were estimated with 1000 replications and the tree was rooted using the midpoint rooting method. Orthology and paralogy relationships were inferred from the resulting phylogenetic tree.

### Gene conversion

The four clusters of paralogs identified for the guinea pig, horse, rat and mouse were tested for interlocus gene conversion, i.e. nonreciprocal transfer of genetic information between genes of the same locus, using GENECONV version 1.81 [21], which is a widely used method for detecting partial gene conversion [50]. Each subset alignment was analyzed using the Clustal W algorithm [47] to search for pairs of sequences sufficiently similar to suggest gene conversion events. Three *p*-values were calculated and compared to assess the significance of the results. Evidence for gene conversion was strong when a fragment had a *p*-value < 0.05 for at least two different types of statistical tests. In each alignment, indels and missing data were treated as a single polymorphism. All polymorphic sites were tested for evidence of gene conversion using adjusted mismatch penalties of 0, 1 or 2, to enable detection of both ancient and recent gene conversion events.

### Evolutionary analyses

To investigate selective pressure, we used the CODEML application in the PAML package version 4.4 [24], which allows the ratio dN/dS to vary across codons and estimates the probability for each codon to be under positive selection. The alignments resulted from Clustal W [47] and PAL2NAL [51].

#### Study of selective pressure in the SAL1 family

To determine if selective pressure varied among sites in the SAL1 family, we used site models implemented in PAML [52], which allows the ω ratio to vary among sites [52,53]. Like for reconstruction of the phylogenetic tree, the chimpanzee sequence (the shortest sequence) was removed in order to have the most possible informative sites. We used three pairs of models including M1a (nearly neutral: 0 <*ω*_*0 *_<1 and ω_1 _= 1) versus M2a (positive selection: 0 <*ω*_*0 *_< 1, *ω*_*1 *_= 1 and *ω*_*2 *_>1) [52], M8a (beta &*ω*_*s *_= 1: 0 <*ω *< 1 and *ω*_*s *_= 1) versus M8 [54] and MEC (a combined mechanistic-empirical model implemented in the Selecton server, http://selecton.tau.ac.il/index.html) [25,55] versus M8a and the PhyML generated tree for the analysis. Likelihood ratio tests were used to compare log likelihood values for M1a vs. M2a and M8a vs. M8 [52]. The Akaike information criterion (AIC_c _score) was used to compare M8a and MEC [55]. Bayes Empirical Bayes (BEB) method [56] implemented in PAML was used to estimate posterior probabilities of selection on each codon, probabilities > 0.95 were considered significant.

#### Study of selective pressure on species and paralogs

To determine whether different species underwent selective pressure, we used the branch-site models of PAML [27,57], which estimate different dN/dS values among branches and among sites. These models can detect a short episode of positive selection if it occurs in a small fraction of amino acids. We tested 13 branches as the foreground branch (i.e. the branch for which positive selection is allowed), eight branches leading to a species (pig, dog, rabbit, macaque, human, gorilla, marmoset and elephant) and five internal branches situated after speciation and before duplication events (in cow, horse, guinea pig, rat and mouse). Figure 3 shows which branches on the phylogenetic tree were tested for positive species selection. We tested each individual branch that led to a paralog in order to detect selective pressures following duplication events. We also used the PhyML generated tree for the analysis. Two models were used to test for positive selection, one model called 'alternative' in which the foreground branch may have some sites under positive selection, and one model called 'null' in which the foreground branch may have different proportions of sites under neutral evolution than the background branch. For the 'alternative' model, three classes were defined: ω0: dN/dS < 1, ω1: dN/dS = 1 and ω2: dN/dS≥1, while in the 'null' model, ω2 was fixed to 1. Like for the site model, LRT [52] and BEB [56] were used.

### Putative function of positively selected sites

To assess the functionality of positively selected sites, the sites were positioned on the SAL1 structure (PDB: 1GM6[13]) and their positions evaluated against the accessible surface area (ASA) of amino acids in SAL1 as determined by ASAView [58]. SAL1 androstenol and androstenone binding sites were previously determined by Spinelli et al. [13]. These amino acids were positioned on the SAL1 structure. Molecular graphics images were produced using the UCSF Chimera package [59].

## Authors' contributions

CM performed the main data collection and analyses. GP provided advice on bioinformatic analyses. IC performed protein structural analyses. CM, FB and PNLM performed mutagenesis experiments and protein production. PM designed the study and helped guide the general analyses. All authors read and approved the final manuscript.

## Supplementary Material

Additional file 1 **Table S1 - Identification of SAL orthologs and co-orthologs**. This table summarizes access numbers of SAL orthologs and co-orthologs, and gene locations in genomes. Genes involved in gene conversion events are also indicated. **Figure S1 - Putative pseudogenes **Evidence for putative pseudogenes in mouse lemur, bushbaby and orangutan are indicated. Click here for file
