# Supplementary material for: The evolutionary history of the SAL1 gene family in eutherian mammals
Source: BMC Evol Biol. 2011 May 28;11:148. doi: 10.1186/1471-2148-11-148 (PMC3128046; doi:10.1186/1471-2148-11-148)
Supplement: Additional file 1 — Table S1 - Identification of SAL orthologs and co-orthologs. This table summarizes access numbers of SAL orthologs and co-orthologs, and gene locations in genomes. Genes involved in gene conversion events are also indicated. Figure S1 - Putative pseudogenes Evidence for putative pseudogenes in mouse lemur, bushbaby and orangutan are indicated. [file 1471-2148-11-148-S1.DOC]

**Additional file 1**

**Table S1 – Identification of SAL orthologs and co-orthologs**

All access numbers refer to NCBI and Ensembl websites. Gene location was determined on MapViewer website from NCBI (http://www.ncbi.nlm.nih.gov/mapview/) [45] and Ensembl website (http://www.ensembl.org/index.html) [46]. Genes involved in gene conversion events are indicated in bold.

## Figure S1 – Putative pseudogenes

Putative pseudogenes were identified by tBLASTn. The traces of pseudogenes with stop codons are listed below. Stop codons are in red and are highlighted in gray. Syntenic information supports the existence of pseudogenes in these genomes.

***Mouse lemur (Microcebus murinus)***

The tBLASTn search was performed using the mouse Mup4 protein sequence against the mouse lemur genome.

Alignment score : 182

E-value : 1.8e-15

Alignment length : 155

Percentage identity: 36.13

Query: 24 QNLN-VEKINGEWFSILLASDKREKIEEHGSMRVFVEHIHVLENS-LAFKFHTV-I-DGE 79

Q+ + V +++G+W+SI LASD +EKIEE+GSMRVFVE I+VLE+S L FK HT+ D

Sbjct: 234745 QSFSYVLQLSGDWYSIYLASDNKEKIEENGSMRVFVERIYVLEHSSLYFKLHTM*****A*****DFF 234566

Query: 80 CSEIFLVADKTEKAGEYSVMY-D-GF-NTFTILK-TDYDNYIMFHLI-NEKDGKTFQLME 134

++ ++ + G Y V + GF +T L + FH N + L+E

Sbjct: 234565 FIDLGVI-NAVLLHG-YIV*****WLSLGF*****HTHH-LNGVPCTQ*****VSFHPSPN-LP-LS-HLLE 234404

Query: 135 LYGRKADL-NSDI----KEKFVKLCEEHGIIK-EN 163

+ N +I KEK +K E +G+ K N

Sbjct: 234403 P-PMLI-IPN*****EIF*****LGKEK-LK-AE-NGL-KYTN 234317


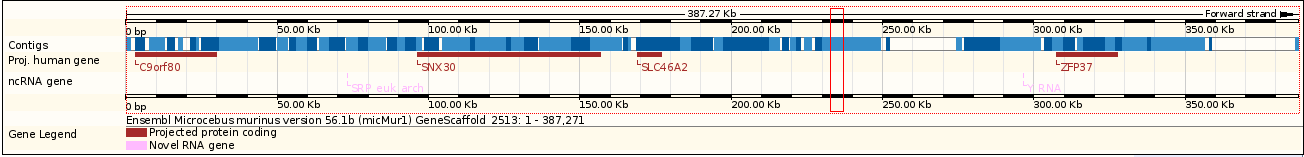


***Bushbaby (Otolemur garnettii)***

The tBLASTn search was performed using the mouse Mup4 protein sequence against the bushbaby genome but no stop codons were identified.

Alignment score : 154

E-value : 1.1e-21

Alignment length : 47

Percentage identity: 63.83

Query: 30 KINGEWFSILLASDKREKIEEHGSMRVFVEHIHVLENS-LAFKFHTV 75

+I+G W+SILLASD +EKI+E+GSMR+FVE I L+NS L FK+HT+

Sbjct: 96740 QISGGWYSILLASDHKEKIKENGSMRIFVEQIQALKNSSLYFKYHTL 96600


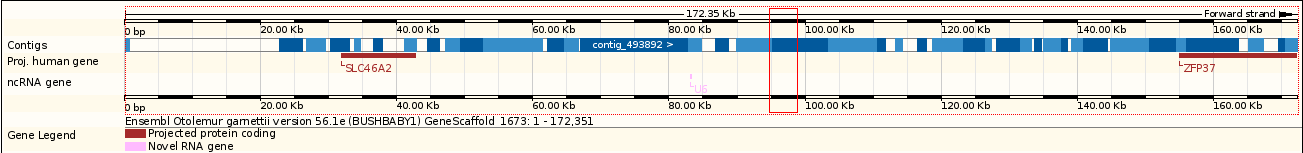


***Orangutan (Pongo pygmaeus)***

The tBLASTn search was performed using the macaque XP_001099373.1 protein sequence against the orangutan genome.

Alignment score : 233

E-value : 1.7e-23

Alignment length : 121

Percentage identity: 48.76

Query: 23 VTS-NFDLSKISGEWYSVLLASDCREKIEEDGSMRVFVEHIDYLGDSSLTFKLHEM-THY 80

+T ++ L +ISGEWYSVLLASD REKIE DGSMRVFV+HIDYL +SSLTFKLHEM +

Sbjct: 109433108 LTQWSY-LLQISGEWYSVLLASDRREKIE-DGSMRVFVKHIDYLRNSSLTFKLHEM*****V-W 109432938

Query: 81 IPPQHFCLGTQWVPFIFPSVWSPVPSFASMTNTQDMAVTVTLPTARTPDVSSQLKERFVK 140

P F +G + + VWS + S+ QD+ + + L + T VS+ K F K

Sbjct: 109432937 -P---FLVG-EGKTEAW--VWS*THTH-SLI--QDL-MDLGL*DSETR-VSN--KN-F-K 109432806

Query: 141 Y 141

+

Sbjct: 109432805 F 109432803


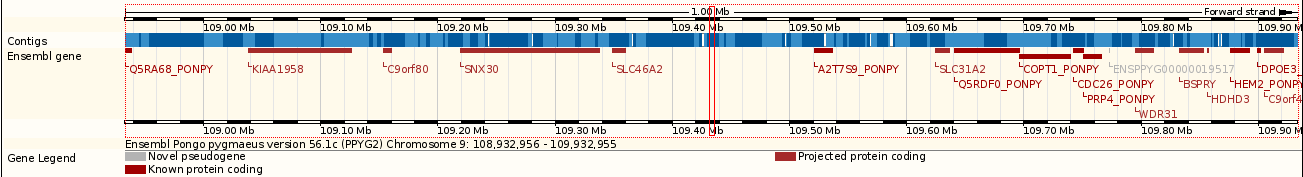


In other mammalian species, no significant match or no syntenic confirmation enabled accurate identification of pseudogenes.
